# *“You know, we can change the services to suit the circumstances of what is happening in the world”:* a rapid case study of the COVID-19 response across city centre homelessness and health services in Edinburgh, Scotland

**DOI:** 10.1186/s12954-021-00508-1

**Published:** 2021-06-12

**Authors:** Tessa Parkes, Hannah Carver, Wendy Masterton, Danilo Falzon, Joshua Dumbrell, Susan Grant, Iain Wilson

**Affiliations:** 1 Salvation Army Centre for Addictions Services and Research, Faculty of Social Sciences, University of Stirling, Stirling, Scotland, UK; 2 Faculty of Social Sciences, University of Stirling, Stirling, Scotland, UK; 3 The Salvation Army, Homelessness Services Unit, Edinburgh, Scotland, UK

**Keywords:** COVID-19, Pandemic, Homelessness, Substance use, Drugs, Alcohol, Harm reduction, Scotland

## Abstract

**Background:**

The COVID-19 pandemic has necessitated unprecedented changes in the way that health, social, and housing services are delivered to individuals experiencing homelessness and problem substance use. Protecting those at high risk of infection/transmission, whilst addressing the multiple health and social needs of this group, is of utmost importance. This study aimed to document the impact of the COVID-19 pandemic on individuals who were experiencing homelessness in one city centre in Scotland, and how services adapted in response.

**Methods:**

Semi-structured interviews were conducted with individuals with lived/living experience of homelessness (*n* = 10), staff within onethird sector service (*n* = 5), and external professionals (*n* = 5), during April-August 2020, using a rapid case study design. These were audio-recorded, fully transcribed, and analysed using Framework. Analysis was informed by inclusion health and equity-orientated approaches to meeting the needs of people with multiple and complex needs, and emerging literature on providing harm reduction in the context of COVID-19.

**Results:**

Those with lived/living experience of homelessness and problem substance use faced a range of additional challenges during the pandemic. Mental health and use of substances were affected, influenced by social isolation and access to services. A range of supports were provided which flexed over the lockdown period, including housing, health and social care, substance use treatment, and harm reduction. As well as documenting the additional risks encountered, findings describe COVID-19 as a ‘path-breaking’ event that created opportunities to get evidence into action, increase partnership working and communication, to proactively address risks.

**Conclusions:**

This rapid case study has described the significant impact of the COVID-19 pandemic on a group of people experiencing homelessness and problem substance use within one city centre in Scotland and provides a unique lens on service/professional responses. It concludes with lessons that can inform the international and ongoing response to this pandemic. It is vital to recognise the vision and leadership that has adapted organisational responses in order to reduce harms. We must learn from such successes that were motivated both by compassion and care for those vulnerable to harms and the desire to provide high-quality, evidence-based, harm reduction services.

**Supplementary Information:**

The online version contains supplementary material available at 10.1186/s12954-021-00508-1.

## Introduction

### People who experience homelessness, multiple morbidities, and alcohol and drug problems

People who are homeless who use substances (illicit drugs and/or alcohol) often experience extreme health inequalities and a wide range of mental and physical health conditions [1], with ill-health equivalent to those aged over 85 years [2]. Despite higher levels of substance use, and poorer mental and physical health compared to the general population [3], many people who experience homelessness access healthcare services at crisis point [4], often using accident and emergency services instead of primary care [5]. This could be for a number of reasons including negative previous experiences of such services, lack of coordination between services, cost of medication, other priorities such as shelter and food, challenges with appointment times, and complex administrative forms [6]. Access to psychological services and treatment for complex trauma, depression, anxiety, and other mental health problems can be extremely challenging for this group, particularly those who also use substances, due to, for example, lack of appropriate services, difficulties with medication compliance, expectations around abstinence, and stigma [7, 8]. Despite this, many do engage with primary care services, including specialist homelessness general practitioners (GPs) [4], but attitudes towards health and social services and the complexity of their needs influences this engagement [2, 9]. Those who struggle with engagement with healthcare services are more likely to experience mental health problems and are at increased risk of death [10].

People who experience homelessness have an increased risk of problem drug and alcohol use [6, 11–13]. Use of illicit drugs and alcohol is common [14]. Problem alcohol use has significant detrimental effects on health and society, affecting more than 280 million adults worldwide [15]. In 2018, there were 7551 alcohol-specific deaths in the UK, with Scotland having the highest rates overall [16]; 1020 deaths were registered in Scotland in 2019, a reduction of 10% from 2018 [17]. People who are homeless who use alcohol are at increased risk of a range of acute and chronic harms including poisoning, seizures, liver disease, cancers, assault and injuries, putting them at high risk of premature death [18].

The 2019 World Drug Report estimated that in 2017 there were over 47,000 opioid overdose deaths in the USA and approximately 4,000 in Canada, an annual increase of 13% and 33% respectively [19]. In the 2020 World Drug Report, the number of opioid-related deaths in the USA reportedly decreased slightly to 46,802, but increased in Canada to 4,398 [20]. It is estimated that there were at least 8,300 overdose deaths, primarily opioid-related, in European Union countries in 2018 [21]. In 2019 there were 4,393 drug-related deaths registered in England and Wales [22]: the highest ever recorded and the largest annual increase at 16%. Drug-related deaths in Scotland are now more than double the number recorded a decade ago. Statistics for 2019 have not yet been released for Scotland, however, in 2018 there were 1,187 drug-related deaths, the highest ever recorded and an increase of 27% from 2017 [23]. There is a higher rate of infections among people who inject drugs, including HIV, and drug-related deaths amongst people who are homeless [6, 24, 25]. Drug-related deaths of people who were homeless in England and Wales increased by 52% between 2012 and 2017 [22], and figures for Scotland using matched data from June 2001-November 2016 showed that 23% of deaths among the ‘ever-homeless’ cohort of individuals were due to drug-related conditions, which was also the largest subcategory cause of death [24]. Harm reduction is therefore essential for people experiencing homelessness and the range of health concerns described above, given they are exposed to more harms, have far fewer protective factors in place such as treatment and stable accommodation, and are generally less able to protect themselves from harm [6].

A range of harm reduction services for those experiencing homelessness are recommended, including naloxone distribution, opioid substitution treatment (OST), managed alcohol programmes (MAPs), education, safe housing such as Housing First, peer-to-peer support, community activism, psychological supports, relational approaches, and advocacy [12, 26, 27]. Person-centred, coordinated, and integrated service models of care for people with multiple and complex needs are increasingly viewed as required to improve quality of life, health and wellbeing, and to reduce harms [6, 28–30]. Assertive community-based outreach is also recommended [18, 31]. A meta-ethnography conducted on effective substance use treatments for people experiencing homelessness highlighted, amongst other components, the importance of staff who are compassionate and non-judgemental, and the need to provide treatment that is long enough in duration to facilitate stability, underscoring how essential it is to understand the complexity and challenge of people’s lives when delivering treatment [32]. While there is no space here to describe the particular additional challenges faced by sub-populations within the wider group of people impacted by homelessness, it should be noted that women who are homeless are more likely to have multiple and complex needs, including being a parent of dependent children, to have experienced domestic and gender-based violence, and have mental health challenges, and these factors should be taken into account when considering provision of harm reduction, housing and health-related services [1, 6, 33–36].

### Impact of COVID-19 for people who experience homelessness and problems with substances

Barriers to healthcare and appropriate substance use treatment for people experiencing homelessness and problems with substances can lead to delayed or even no treatment which has the potential to be particularly problematic in the context of COVID-19. COVID-19 is a disease of the respiratory system [37] and, while everybody is at risk of infection, people who are homeless and use substances are at significant risk of being negatively affected due to increased likelihood of underlying health conditions making them a high-risk group [38]. Additional problems relative to the pandemic may include the inability to shield or isolate oneself due to not having safe housing; poorer access to resources to protect health (such as personal protective equipment, PPE); potential medication shortage; disruption to community pharmacy dispensing and harm reduction services such as injection equipment provision (IEP); lack of access to preferred substances due to changes in illicit drug markets or changes to type and mode of administration of substances, including lack of access to alcohol; increased dangers of withdrawal; increased stigma and discrimination due to having to break lockdown restrictions; and reduced access to treatment for problem substance use [39–42]. For people with problem alcohol use, there is a risk of withdrawal if they cannot maintain their supply which can lead to serious health consequences and even death. They might also substitute alcohol with illicit drugs [14, 43] which can increase harm, for example by being unaware of ways to reduce overdose risk such as use of naloxone or as a result of unsafe supply. There are additional risks associated with retraction of informal support service from friends, families and communities due to social distancing measures.

### Impact of COVID-19 on harm reduction services for alcohol and drugs

The increased likelihood of substance-related harm, and death, that the COVID-19 pandemic has brought to an existing crisis of substance harms has been referred to as a dual public health emergency, with immediate action being called for to prevent the spread of infection amongst an often immune-compromised population [44]. It is essential that services are maintained and, where necessary, altered to meet the differing needs of people who use substances in response to the pandemic, and that funding for such services is safeguarded [45, 46].

Globally, this has included changes to drugs harm reduction services, including: designating harm reduction services to be essential [46]; developing emergency harm reduction plans [46]; access to COVID-19 screening and testing [47]; changes to service and medication provision to comply with pandemic guidance [41, 42, 46–56]; improved access to naloxone and IEP [47, 50]; increased awareness of the need for clean water for injecting [57]; general guidance about reducing COVID-19 spread in services [58–60]; the need for a ‘safe supply’ of drugs [46]; and the need for holistic models of care that attend to mental and physical health and housing needs [46]. While evidence is still emerging on how the pandemic impacted drugs harm reduction, strict lockdown rules have appeared to reduce the number of people accessing IEP and other harm reduction services [61, 62]. Other public health guidelines, such as social distancing, maintaining high levels of personal hygiene including regular hand washing and the wearing of masks, place additional demands on people who use substances, which may hinder the provision of effective services [63]. There is also some evidence that social distancing measures have been linked to increases in drug overdoses because people have been injecting on their own more often [64].

For alcohol, provision has been more limited but has included access to withdrawal management medications [44]; safer drinking tips [65]; clear guidance for health care providers [66]; and implementation of MAPs [67]. There have been challenges maintaining alcohol-related services during the pandemic, with many reducing resources and hours, or shutting completely which, when added to existing co-morbidities and risk environments, may have severe consequences, especially for people who are homeless [53].

### Changes to services in the UK during the COVID-19 pandemic

There have been a wide range of changes to services across the UK. The majority of services that support people facing multiple disadvantages, such as people who are homeless and use substances, have faced pressure to rapidly adapt services to meet needs during a period of unpredicted crisis. The need for increased IEP services is essential, as research has shown that access to injecting equipment has decreased rapidly during lockdown across England [62]. Home delivery of injecting equipment has been implemented, as well as increased encouragement of peer distribution [68]. Research has shown the need for further equipment to be readily available, such as safe inhalation equipment for crack cocaine [69]. Other responses have included: rapid rehousing in hotels for people who were homeless which allowed people to self-isolate effectively [70]; the introduction of medication delivery for prescriptions and controlled drugs [71]; naloxone home delivery [68]; an increase in treatment for opioid dependence and changes to the ways that treatment was delivered e.g. reduced waiting times and less frequent prescription pick-ups [72]; outreach support [73]; and clear guidance for commissioners and providers of services for people with problem drug and alcohol use [74].

In Scotland, there were a number of changes made to service landscapes though these naturally differed markedly across the country [75; Fletcher, personal communication; NHS Greater Glasgow and Clyde Drug Trend Monitoring Group, personal communication]. This study is focused on the city centre of Edinburgh, and the changes described in this paper do not characterise a Scotland-wide approach, nor do they provide an account of the whole city’s response. Edinburgh has the second-highest number of homeless households in Scotland in 2019/20, with 3355, which was a 5% increase from the previous year [76]. Because the response to the COVID-19 pandemic will differ locally as well as nationally [37, 77, 78] it is necessary to explore neighbourhood level responses to gain learning on what changes were made and how successful they were. This can inform national and international as well as local responses. The Scotland-wide changes that are documented and relevant to highlight briefly here include: people who were homeless were rapidly rehoused in hotels [79]; individuals no longer had to use the local choice-based lettings system for new social housing tenancies [79]; the Scottish Government introduced The Coronavirus (Scotland) Act 2020 as an emergency law to protect all renters from being evicted from their accommodation [80]; a directive was issued to health and related Boards regarding the importance of maintaining service-level provision of drug and alcohol services and protecting staff from redeployment to ensure harm reduction services could be maintained [81]; increased assertive outreach by medical staff including home visits [75]; increased IEP and OST, for example through postal delivery [82]; rapid access to OST [38]; increased naloxone provision as a result of the Lord Advocate issuing a statement of prosecution policy in relation to the distribution of naloxone by non-drug treatment services who were registered with the Scottish Government [83]; and recommendations for alcohol services and advice for people with problem alcohol use produced by Scottish Health Action on Alcohol Problems (SHAAP), although this was not specific to people who are homeless [84].

### Aim, rationale and interpretive frameworks for the study

As described above, substantial changes have been observed worldwide in relation to service provision for people experiencing homelessness and problem substance use in response to COVID-19 and associated public health measures. There is, however, a substantial knowledge gap regarding how those closely involved, such as staff and people with lived/living experience, have experienced changes. This study was designed to specifically address this gap. For pragmatic reasons, we utilised a rapid case study design [39] and focused data collection on onethird sector (not for profit) service (the Wellbeing Centre) that we had existing research partnerships with via a Community-University partnership called the Salvation Army Centre for Addiction Services and Research at the University of Stirling.

The Wellbeing Centre is a drop-in service for people who are, or are at risk of being, affected by homelessness, run by The Salvation Army (described as ‘the Centre’ throughout). The Centre supports people holistically, in all aspects of their lives, not just their housing and homelessness needs. Although it is not a service specifically for people with problem substance use, many of those attending the Centre use drugs and/or alcohol and additionally experience a range of mental and physical health problems. The Centre has a strong harm reduction ethos and provides a café, shower facilities, and various groups and social activities. The provision of harm reduction services changed in a range of ways in response to the pandemic, as detailed in our linked paper [85] where our team described the impact of the pandemic on those using the Centre and, most specifically, how the Centre adapted to the needs of their clients. In this current paper, we present our results from participant data concerned with wider issues including the mental health and substance use impacts of the pandemic and lockdown restrictions, and how individuals and organisations worked together across the sector/city centre to reduce risk of harms for those experiencing both homelessness and problems with substances. The research questions addressed in the current paper are: how did the COVID-19 pandemic impact people with lived/living experience of homelessness and problems with substances in city centre Edinburgh; what changes/adaptations were implemented by related services during the pandemic for this group of vulnerable people; and what related challenges/barriers/risks and opportunities/benefits were perceived? In interpreting our data, we draw upon inclusion health and equity-orientated frameworks [86–89], alongside international literature on providing harm reduction services in the context of the COVID-19 pandemic.

Inclusion health aims to prevent and effect social and health inequalities for marginalised populations, including those experiencing homelessness and problem substance use [18, 86]. The focus is on increasing awareness of the effects of extreme inequity, the need for prevention and early intervention, and improving access to services for these populations [18]. Inclusion health approaches have been taken up as a way of proactively addressing the needs of marginalised groups and include interventions such as integrated mental and physical healthcare, low threshold harm reduction, and provision of housing [18]. Relatedly, equity-oriented approaches, a term typically used in Canada, aim to reduce the health inequalities experienced by marginalised populations and improve their health through structural and policy changes [87–89]. Both inclusion health and equity-oriented approaches can include violence and trauma-informed care, and emphasise the need for partnership working and coordinated/integrated care [18, 87].

## Methods

The methods are described in more detail in our linked paper [85] and are therefore briefly summarised below.

### Approach and ethics

A qualitative exploratory study using a rapid case study approach [39] with semi-structured interviews was conducted between April and August 2020. Ethical approval was granted by University of Stirling’s General University Ethics Panel (paper 899) and the Ethics Subgroup, Research Coordinating Council of The Salvation Army (RCC-EAN200504). Rigorous risk assessments were conducted for face-to-face data collection, as per organisational protocols.

### Participant recruitment

Participants were people with lived/living experience of homelessness who used the Wellbeing Centre; service staff/managers of the Centre; and wider stakeholder/external professionals who worked closely with the Centre, to ensure that data included diverse vantage points. Purposive sampling identified individuals based on role/membership of these identified sampling groups, and gender, to ensure the sample reflected a wide range of views and experiences. All participants were provided with a participant information sheet and an opportunity to ask questions, with 48 h ‘cool off’ periods.

Informed consent was granted at the beginning of each interview. All interviews were audio-recorded with consent and lasted an average of 38 min. The interviews were conducted by two researchers: WM conducted staff and stakeholder interviews and JD conducted the lived/living experience participant interviews. All interviews were conducted via telephone for staff and stakeholders. Lived/living experience participant interviews were either conducted via telephone or in-person in the Centre, to provide choice. In-person interviews were possible as JD was working in the service throughout the lockdown period, with required health and safety risk assessments undertaken. After each interview, participants were provided with a debrief sheet which gave further information about the study and support available. Detailed field notes captured researcher reflections to enhance reflexivity [90].

### Data analysis

Data were transcribed in full and, where relevant, used local Scottish dialect (see Additional file 1 for a glossary), and analysed using Framework [91] in NVivo 12. Framework is suited to policy- and practice-relevant research by providing a structured and transparent approach. More detail regarding data analysis of the transcripts and documents can be found in our linked paper [85]. Additional file 2 provides a glossary of terms used in the study and Additional file 3 includes a list of abbreviations used.

## Findings

A total of 20 interviews were conducted with 10 Centre service users with lived/living experience of homelessness and histories of/current substance use concerns, five Centre staff members, and five professionals working in the wider service sector. Pseudonyms are used throughout. Table 1 provides participant characteristics.

**Table 1:** Participant characteristics

Stakeholders (*n* = 5) Mixed gender***—***details removed to protect identity	
*Third sector (not for profit) organisations*	3
*National Health Service (NHS)*	1
*Commissioning*	1
Staff (*n* = 5) Mixed gender—details removed to protect identity	
Service users/people with lived/living experience of homelessness (*n* = 10)—two women and eight men—eight people were vulnerably housed at time of interview with two people having more housing stability but still requiring support	

Data are organised into three overriding themes: the impact of COVID-19 on people who were experiencing homelessness and problems with substances, in particular the mental health implications and use of drugs and alcohol; the challenges of organisational and city responses to these needs; and opportunities presented by the pandemic. While our linked paper [85] presents data focused on the Centre’s organisational response to the pandemic, this current paper takes a wider perspective by providing insights into how the pandemic impacted individuals with multiple and complex needs, and the city centre’s response to COVID-19 more broadly. The three overriding themes are now presented below.

### Impact of COVID-19 on people who were experiencing homelessness and problem substance use in Edinburgh city centre

Participants with lived/living experience discussed facing a raft of substantial existing challenges in their lives relating to mental health, losing care of children, isolation from family, social exclusion, substance use, disengagement from mainstream services, and imprisonment, amongst others. As described in our related paper [85], the lockdown period in general, but particularly the very early lockdown period, was characterised by feelings of confusion, anger, loss, and fear, which further exacerbated the social isolation that many experienced prior to the pandemic.

Participants with lived/living experience discussed struggling with their mental health, problems that pre-existed, and were exacerbated by, the lockdown, with life described as a “*constant struggle*” (Steven). All interviewees with lived/living experience described their mental health difficulties as being aggrevated by the lockdown, as access to support became substantially limited alongside increased social isolation. The need to shield added to the acute feeling of loneliness and social isolation during the lockdown period, as described by Maria:

*Aside from not being allowed to go out the fucking door aye. I’m not allowed out. Everybody else can go for a walk, I am imprisoned in the square. I’ve walked around the block and avoided everybody all the way, and I’d have a mask on.*

As well as Maria, a number of other interviewees with lived/living experience discussed having to shield (physically isolate) as a result of being in a ‘high-risk’ category:

*Because I’m type one diabetic, pal, asthmatic, and it goes for your lungs.* (Frank)

*I have COPD [chronic obstructive pulmonary disease] so I was listening to the Government and I decided to shield myself for the twelve-week period. For ten weeks now.* (Andrew)

Jacqui described the lack of support provided in her hostel accommodation, due to a lack of staff, and the negative effect this had on her:

*They are not even doing room checks… basically no support.*

Owen discussed being accommodated in a hotel during lockdown where he noted the atmosphere lacked companionship and friendship. Although receiving support whilst staying in the hotel, he said that he would tell people he was fine because he did not feel comfortable sharing his true feelings:

*Folk have just like sort of isolated themselves. I know it’s the thing to dae, it is one of the rules to keep yourself to yourself. I just felt I was keeping myself away fae everybody. Yeah, I just felt, because there was naewhere for support, I just felt I was shutting myself away a lot in the bedroom and I was getting really depressed quite quick. And anybody I was meeting I was just keeping it short and sweet and basically just lying to the person I was maybe speaking to. And that’s not how I was feeling. I was just saying ah I’m alright, I’m good, even though I wasnae… I was actually really depressed, and I just wanted somebody that I could share that with.* (Owen, Client)

Although some of the city services had stayed open and set up phone support and online groups, these did not seem to be sufficient on their own to offset the increased levels of social isolation being experienced. Some people described not having a family network to fall back on in such challenging times, making the social isolation all the more acute. Wayne talked about his struggle with the sudden removal of social interactions and consequent isolation:

*I’ve not got a wife, I’ve no kids you know. I’ve got one parent left, I’ve got a brother who’s got his own wife and daughter who… I mean when that happens, you’ve probably seen it yourself, you know. They more or less just drift into their own family. They see you from time to time but that’s it. I am not blaming anybody. If it’s anybody’s fault it’s just mine, just because I’m a bit too shy.*

Overall, the lockdown seemed to have been a mixed period for the use of drugs and alcohol. Some participants stopped use completely or reduced, others were able to maintain their recovery, whereas for some individuals their use continued to be heavy or increased. John illustrates how his substance use reduced during the pandemic:

*I’ve not had a drink for a month now. I’ve not had a line of cocaine for about four weeks as well, and I’ve managed. I’ve done a little rattle off the Valium as well, almost three weeks now, it will be three weeks tomorrow. I was taking a lot of the street valium (illicit benzodiazepine of unknown strength) for a few months there and it was a generally a good thing at nighttime. I was generally just taking them to sleep at nighttime, you know. I was taking them before my bed but you hear all these cases of people you know like dying from it and all that like and, I suppose, during the lockdown, I realised I don’t want to be one of those people. So, I’ve been three weeks off the Valium as well. And I’ve been off my antipsychotic medication for a while as well. It’s been a couple of weeks but I definitely feel a whole lot better in myself.*

Similarly, Wayne described making a conscious effort to gradually reduce use of both alcohol and drugs. For him, reading was a key form of escapism which allowed him to stop thinking about substances and manage temptation. He described this as a need to “*grow up a bit*”, and “*make the most*” of his situation. Alternatively, Owen described shutting himself away and consuming large quantities of illicit substances. Wayne described needing more support for his depression and anxiety at the beginning of the pandemic, and highlighted the impact poor mental health had on his substance use:

*Basically, depression and anxiety are the things that are the gateways, I think, to drinking but, you know, people sort of want to point out the main problem first, I think, than deal with the underlying issue you know.*

Andrew described heavy use of cannabis over the period, “*more so than ever before*”, although he commented that this was not something he needed support with, and Chris described an increased use of alcohol:

*Just drinking mare… It sort of went up, then down, then up. Then I started drinking vodka and all that. And me and vodka… it just equals fucking jail… relationships, problems, shit.*

Steven described general problems with the use of alcohol and Valium:

*Alcohol is really bad. Same with Valium by the way. I blank out on it. It takes over me. I wake up the next day, “You done this, you done that, you done that”. That’s not me man, that’s the worst thing about it. But you are the one that took the drugs, you are the one that gets the blame.*

Jacqui described using a large quantity of ‘street valium’, but also expressed her desire to stick to the prescription she received at the pharmacy (chemist) because of concerns about unsafe supply:

*I’ve got to take like 50, 60, ken what I mean? But the ones that have been going about have only been needing maybe like 40. I’m just wanting to try and stick to my ones that I get from the chemist, even though it’s nothing, but if I can just stick to my chemist*.

For those who reported increasing their substance usage, this seemed at least in part due to the emotional challenges caused by lockdown, the isolation, and diminished availability of support.

People with lived/living experience talked about mixed experiences of changing drug supply in the city during the pandemic. Some reported no changes, others felt that drugs had become more expensive or more difficult to access, whereas some participants described drugs being more readily available and more people using them.

*Maybe a gram of grass, like it used to be a tenner, it’s now twelve fifty. Xanax are, they were two pound, they are now two pounds fifty, plus like I’ve seen this happen before in droughts, when the price goes up, it doesn’t come down.* (Wayne)

*There is more of it… It’s getting stronger. Actually,* *there is a lot more of everything.* (Steven)

*Probably a lot more sales though. A lot more folk taking drugs, probably over this period than any other period, to be honest. I’ve started using more cocaine, just to try and get us out and about, and get the depression away.* (Owen)

Staff participants also talked about changes in drugs supply and increased use of harm reduction services, such as IEP, during the pandemic:

*We heard that the drugs were drying up but I have to say the Valium situation has become a lot worse. So people taking vallies (street benzodiazepines) is a lot worse. But there has been a lot more uptake on the needle exchange than we ever would have expected so that was good.* (Samantha)

Martin (Staff) reflected on his perception of an increase in uptake of OST, particularly among people who were unable to buy drugs due to a lack of income from begging.

Yet, in spite of these complex and intersecting challenges, or perhaps as a result of them, individuals discussed a strong desire for positive change in their lives. For some, this positive change was envisaged as reducing, or completely stopping, using drugs and/or alcohol. Participants who specified this were at different stages of the process. John described having not used drugs or alcohol for approximately three or four weeks. He described having previously relapsed due to a difficult relationship break-up but now wished to make a positive change in his life.

### Responding to the needs of people experiencing homelessness during the pandemic

#### Housing support

The housing of people who were on the streets, and provision of housing support for people in temporary and supported accommodation, were affected differently by the pandemic. The rapid rehousing of people who were deemed street homeless across the city was described as a “*massive triumph*” (Naomi, Stakeholder), and praised by staff, stakeholders, and people with lived/living experience alike. There was a view that the support provided to people to quickly move them from the streets to hotels was positive, and had facilitated engagement with other services and supports:

*They were actually pretty quick in terms of putting extra accommodation in place so that nobody needed to rough sleep. And that, for a proportion of our service users, made a difference. They had somewhere to go, they wouldn’t be rough sleeping. If you got thrown out of a hostel you wouldn’t need to hope that there was a space left in the night shelter.* (Martin, Staff)

*I have some acquaintances. They are finding it (rapid rehousing) quite good, it’s a positive experience yes, yes. I’ve spoke to a couple of guys who stay on the streets quite regular and yeah it’s been positive.* (Andrew, Lived/Living Experience)

This rapid rehousing allowed services to identify new people to work with and support those such as ‘sofa surfers’, those who had become newly homeless, and those without recourse to public funds via a specialist migrant worker. Participants also discussed concerns that they had relating to the rapid rehousing, for example in having many people with complex needs in one hotel:

*It’s what’s to be expected… if you put seventy, eighty people, all of whom have their own issues, and put them all tae live in the same place you know it’s nae surprise that some of them didn’t manage. It’s nae surprise that some have ended up being victims of others, and it’s how we manage that sensitively and dinnae necessarily penalise the ones that have been misbehaving. Because there have been some violent incidents in the hotels. But I would rather live in a city where we can get people off the streets, and then deal with the issues that they bring, rather than just saying “ah we’ll no bother with accommodating anyone because they are just gonnae cause hassle”.* (Brian, Stakeholder)

Participants highlighted what they saw as challenges yet to transpire relating to housing people quickly once the hotels were re-opened as hotels again to ensure that no one would need to go back to being street homeless. Some participants stated that the pandemic had created opportunities to end homelessness that needed to be urgently addressed before the rapid rehousing programme came to an end:

*When we come out of the other side and they have been in a hotel, and they have got used to being in accommodation, some of them will be back on the streets again and that will have quite an impact on folk psychologically. I’m not silly, I know the hotels can’t put them up, but it’s actually been a very good thing that has come out of this.* (Caroline, Staff)

While rapid rehousing was praised, there were issues with ongoing housing support being suspended during the pandemic: people were unable to get the social care support required, most specifically for those living in supported accommodation and in their own tenancies. A lack of PPE also meant that staff and stakeholder participants highlighted being unable to visit people in their homes and provide the support needed:

*We are seeing a lot of people who are in supported accommodation in real crisis at the moment and really struggling.* (Kate, Staff)

Brian (Stakeholder) illustrated the problems that he had encountered in such situations, as he touches on in a quote above, with individuals becoming additionally vulnerable to being taken advantage of or abused by others because of the lack of oversight by professionals:

*A lot of folk that have needed visiting support, or some sort of social care input, have no been able to get it. These are the ones that their tenancies are being taken over by, you know, dealers or whatever. Whether it be cuckooing or other kinda aspects of that. It could just be people no being able to gatekeep as effectively withoot having some sort of supportive relationship ti’ back them up. If we are saying we want services to be able to visit people at their tenancies and things like that, well you need to let us dae that. So, whatever it is, is it gloves, is it face masks, is it regular testing?*

Several participants with lived/living experience reflected on their experiences of housing provision during the pandemic: some described a lack of support in temporary accommodation and poor-quality accommodation, with problems that had not been fixed, however, for Steven, there was finally hope regarding the prospect of being provided with a house:

*I will get a house this year within the next six, seven weeks. I just done my first bids yesterday. And see, as soon as we did, it went tick, tick, tick, instantly. It never happens like that.*

As well as the challenges outlined above, the potential for a surge of homelessness caused by the general instability of the pandemic was also raised:

*There are a lot of new people, from what I gather, in the hotels, that have never really been homeless before, you know, family breakdown. There is increasing young people from family breakdown, from all sharing the same space day after day with no escape… young people have left, they have been asked to leave, and nowhere to go* (Martin Staff).

*What I’ve noticed is there is a lot more homeless now than there was… before this happened, and I think that was because some folk were staying with friends, sofa surfing, or someone in improper accommodations, so I think more have come out of the woodwork now* (Owen, Lived/Living Experience).

#### Access to wider services

As well as changes to housing support, participants described changes to primary healthcare, mental health and substance use treatment, and food provision. Participants with lived/living experience described either not being registered with a GP, or not attending their GP due to feeling judged or discriminated. These were issues that existed before the pandemic, as Chris recounts here:

*Aye there is nowhere to go but it’s also just horrible. My GP doesn’t gi’ a fuck. I don’t even bother. I’ve already told them they look and talk to me like I’m something on their shoe. So I’ve never been back.*

While some of these problems preceded COVID-19, the pandemic was reported by some to have exacerbated them, including: no face-to-face contact with GPs or no access to GPs at all; cancelled appointments with Community Psychiatric Nurses (CPNs); a lack of wound care, sexual health, mental health or dentistry services; and people being turned away if they attended A&E for non-COVID-19-related problems. Several lived/living experience participants described negative experiences from a range of services:

*I’ve still to get registered with a doctor. I wanted to speak to [doctor] but you know s/he seemed to be quite anxious to get me out of his/her hair.* (…) *I had a dentist appointment to get the bottom plate and this was like the third appointment and like they didn’t even bother phoning the clients. They just sort of put on a message, sorry due to the COVID whatever outbreak there will no longer be appointments.* (Wayne)

*More help, more interaction from my CPN because, fair enough right, s/he gets on at me like, they say that we are a team right? Do you ken I’ve not seen my CPN in about six months? And that’s no joke like, I’ve been five, say five months.* (Jacqui)

Brian (Stakeholder) described the need for these services to have a better understanding of the complex lives of people who experienced homelessness and reflected on clinics having fixed appointments and penalising/banning people who failed to attend:

*“No we cannae dae that, that’s not how we dae it”, and “oh you missed that appointment so you are banned”. Let’s stop that. Let’s realise that people who lead these sorts of lifestyles dinnae get to appointments. I mean they try. But they dinnae get to them. So let’s make the services suit the people, rather than find people to suit the services. Let’s, ken, use this as an example of how to change the service to suit the time. You know, we can change the services to suit the circumstances of what is happening in the world.* (Brian, Stakeholder)

A number of participants also noted a lack of services and support for people who used alcohol, rather than drugs, and changes in supply:

*It seemed that the people who were struggling the most were the drinkers rather than the opiate users because, you know, we’d made it easier to access scripts, we’d made it easier to get needle exchange, you know. There was stuff there, you know, the chemists werenae shut. But a lot of the places where folk went for their booze were shut. There was nae people to beg money off for a can. (…) Traditional street drinkers seemed to be the ones that were having the most problems.* (Brian, Stakeholder)

Finally, ensuring that those using services were able to comply with physical distancing rules when using health and related services, and outside of them, was a concern discussed by both staff and stakeholder participants. According to stakeholder interviewees, a reluctance to comply with lockdown rules related to a general ambivalence about the pandemic and risk:

*It’s been an eye opener for the clientele base. It took them weeks and weeks and weeks to realise this is actually here to stay. This is a killer, you know, we are not just saying this (…). It took them a long, long time and I think, just now, we are ten weeks into, nine weeks into it, there is a lot more of them realised that this thing is big.* (Jack, Stakeholder)

### Opportunities presented by COVID-19

As well as these substantial challenges and difficulties posed by the COVID-19 pandemic, participants also discussed a number of opportunities which had arisen from the service adaptations made during this period: an increased ethos and practice of partnership working, and the emergence of new sites for harm reduction provision.

#### Increased ethos and practice of partnership working

There were a range of changes to the way that services were run and these were praised by participants, particularly in relation to increased contact with certain healthcare providers, and easier processes for accessing OST and other medications. In particular, staff and stakeholder participants discussed an increase in multi-agency partnership working and a related reduction in administrative barriers. Kate (Staff) described how, from the early stages of lockdown, there was additional communication between services:

*I can remember, all of a sudden, every time I turned away from my computer there was another ten emails, and there was lots of phone calls. What was really positive was there was a real multiagency approach. It definitely felt that, and there was a lot of positives that came from that too. People were really up for ‘how are we going to change how we are working?’.* (Kate, Staff)

Brian (Stakeholder) explained that, prior to the pandemic, there had been a long-standing sense of competitiveness between agencies which had changed over the weeks of lockdown:

*There has definitely, across the sector, been a lot more appreciation of the value of partnership working now. That might sound daft, like that anyone even had an issue wi’ that, but, certainly, having worked in the homeless field in this sector in Edinburgh for fifteen years, there has often been a bit of competitiveness between different agencies. Especially when a lot of services are put oot to competitive tendering. For all that we, you know, are there to help people, we dinnae always necessarily play nice wi’ each other.* (Brian, Stakeholder)

Brian went on to discuss changes which had occurred during the pandemic which drove services to draw on each other’s strengths and resources, something he hoped would continue with the sector working “*more holistically, rather than a collection of different agencies”*. Echoing this, Naomi (Stakeholder) described COVID-19 as a ‘path-breaking’ event:

*That’s probably one of the biggest things we should learn, you know, the world of homelessness and drugs hasn’t changed for the past twenty, thirty years significantly, up until a pandemic has occurred. And what shall we take away from that? This pandemic has freed up a sort of autonomy to make decisions that previously would have went through years of paperwork to approve.* (Naomi, Stakeholder)

The Centre was seen as central to the city’s joined up approach during the COVID-19 pandemic by working closely with the wider service network to distribute naloxone, IEP, and facilitate rapid access to OST prescribing and multi-disciplinary health outreach services. As described in the linked paper [85], IEP was previously provided by a visiting mobile service, a van parked outside of the Centre. During the early weeks of the pandemic, Centre managers decided to bring this service into the Centre and Richard (Stakeholder) spoke about the positives of this move:

*The IEP became more closely integrated with the rest of the service. They started doing take home naloxone and, best of all, they offered a setting for a low threshold opiate replacement therapy service which was desperately needed at the time. And I am very, very hopeful that this won’t get reversed, but that actually was a rapidly emerging bit of practice that got accelerated very much by the COVID crisis. And they are offering that social care alongside it. It makes it much, much more effective, and practical and safe.*

Richard described how this rapid access to OST and multi-disciplinary outreach had previously been proposed to statutory services but had been held back by a number of administrative barriers. According to interviewees, such barriers were reduced during the pandemic by the realisation that the city was in state of crisis and needed rapid solutions. In addition to internal changes, stakeholders highlighted the role that Centre staff had in playing a coordinating function for wider services. Richard (Stakeholder) stated that the trusting and close relationships that staff had with their service users allowed them to have *“a finger on the pulse”* of what was going on in a rapidly changing situation:

*We knew that we’d have a sudden change for a lot of patients. They [Centre staff] facilitated us doing a survey of the needs of people in the various hostels and day centres, and anybody that needed medical care. (…) They have got a finger on the pulse of what is going on in that population.*

For Richard, the Centre thus became a natural point of *“networking activity”* for wider services.

Related to the emergence of greater partnership working, creativity, and fewer administrative barriers to practice, some described a levelling of the ‘playing field’. Kate (Staff) commented that this proactive networking and communicating across the statutory and non-statutory sectors in the city centre during the pandemic had created a perception that, as a third sector (not for profit) organisation, staff in the Centre could challenge established norms:

*People are really up for thinking about problem solving and trying to be creative. I’ve definitely felt that that has been a real positive too that I would like to continue. A really creative approach and thinking outside the box more as well. And you are questioning things and questioning the norm and asking some questions. As a third sector organisation it maybe didn’t always feel that you could do that, but now it feels because we have been quite creative that we can maybe do that.* (Kate, Staff)

It was hoped that, moving forward, this would present opportunities for third sector organisations to more effectively challenge the norms of established practice.

Naomi (Stakeholder) commented that maintaining a level of face-to-face provision during a period where, across the nation, many other services were switching entirely to phone-based support, was a crucial part of the wider city centre’s homelessness COVID-19 response. Martin (Staff) highlighted that those using the Centre were being able to engage with their CPN far more frequently during the pandemic because of the availability of video conferencing appointments via laptops issued to hostels. In fact, Richard (Stakeholder) had observed a growing perception that frequent short phone contacts were an effective form of support for people with multiple needs:

*Phoning them up every couple of days and just having a chat and seeing how they are doing is becoming more standard practice and that is actually a bit of learning that we are going to get from COVID. Rather than a barrier, actually providing people with phones, but then making sure to keep in touch with them in useful bite-sized contacts.*

Owen (Lived/Living Experience) highlighted an access barrier related to this, however, stating that while many services were available via online methods during the pandemic, they were only available to those who could engage online or by telephone.

#### New sites for harm reduction

Services in the city centre that already had firm commitments to harm reduction prior to the pandemic were viewed as being best able to ‘upscale’ harm reduction during it. In this way the pandemic acted as a catalyst. Being pragmatic and breaking down administrative barriers were viewed by participants as key in this upscaling, as well as the proactive partnership working discussed above. When asked what could have been done better at the start of the pandemic in relation to harm reduction, Max (Stakeholder) believed it was important to understand more about what the resistance was to changing established practice, and being able to explore with those in senior positions what the concerns were, in order to become more solution focused:

*They were very fixated with “this isn’t going to work, this is wrong, we can’t do it this way”. There was no sense of a shared goal, or shared priorities, or shared values even. So you heard that it wouldn’t work but you didn’t hear about what the issue was in all of this. Obviously shared values are essential to all of this.*

As highlighted above, some participants described a lack of harm reduction services for people who were homeless who were using alcohol as their main substance and described the potential of implementing a MAP within the Centre to address this:

*We were looking at the possibility of a managed alcohol programme but that looks very difficult to do as a drop-in in the current circumstances. If that were going to happen on a patient basis, a day service basis, then potentially [Wellbeing Centre] would be a good location for that.* (Richard, Stakeholder)

Participants also reflected on positive changes to the way in which medication as part of OST was prescribed during the pandemic, via outreach models. In order to keep people safer, and ensure they were not going into withdrawal, people were able to access the medication they needed much more quickly and easily. This was facilitated by NHS clinicians being able to access people who needed OST through the Centre’s multi-disciplinary health clinic and other health outreach clinics that were set up within other homeless services, such as hostel accommodation and the rapid access hotels.

“*The GP came actually into the hotel where I am, you could see a doctor in here*.” (Owen, Lived/Living Experience)

*It’s been easier to get people access to scripts. They’ve made it much easier and quicker to get access to a methadone script. Certainly that’s a bit of feedback from a service user. They felt that that was much more helpful and much more like a drug service that they wanted.* (Brian, Stakeholder)

Martin (Staff) described the multi-disciplinary health and prescribing service set up in the Centre as providing significant opportunities for OST to be initiated:

*For quite a few guys, that was maybe the first time they’d even thought about getting onto a prescription. For some of them they had thought about it but the existing process was quite slow and quite scary in terms of, you need to be at particular levels or, if you are doing it through one of the hubs, you need to be X number of visits over X number of months. That just doesn’t work for our client group. So, to be able to turn up on the day, have a discussion with somebody, and walk away that day with a prescription, was just fantastic.*

Several participants with lived/living experience also described having positive experiences of accessing OST, feeling that they had more control over their medication and dose/frequency, and enjoyed the benefits of being able to pick up their prescription directly from the pharmacy, instead of the GP. They also discussed positive changes in terms of moving from daily to less frequent pick-ups, and having very positive experiences with pharmacies:

*I’ve got the best pharmacy in the world. It’s weekly now, but [doctor] actually put it on monthly but I said to my pharmacy “I will lose that or someone will steal that off me and I will end up taking it. I said I am going to end up rattling here.” S/he said “Well I will just give you it weekly”. I swear to you it was like that, “Would you do that for me?”.* (Steven, Lived/Living experience)

There were challenges noted too, however, with regards to having too much medication at home due to longer times between collections, as Wayne describes:

*If I picked up you know like 500mls on Monday, or 700mls to do me until the following Monday, then, you know I’ve got a feeling that I’d probably, I’d abuse it. I just prefer the order of picking it up daily. It gives you a reason to get out the house or maybe go to the shop… I like that routine.*

This section has described a number of important opportunities that arose from the service adaptations to ameliorate risk during this period: an increased ethos and practice of partnership working and the emergence of new sites for harm reduction, which alleviated harms for those at risk from COVID-19 and other health and substance use harms.

## Discussion

This study aimed to provide a window into the COVID-19-related response for people experiencing homelessness and problem substance use which took place within one city centre location in Scotland. The study addresses the substantial knowledge gap highlighted by Vasylyeva and colleagues [92] on the effect of the lockdown on service provision for this group of people highly vulnerable to a range of harms. Effective responses to a global pandemic require local action [90]. Effective scale up also requires responses to be repeatable and ‘generic’. We undertook a rapid case study during the pandemic using interviews with individuals with lived/living experience of homelessness and past/current substance use challenges in the city centre of Edinburgh, and staff involved in providing the COVID-19 response. Our study adds to other publications that have documented the city’s swift and co-ordinated response [75].

The thematic categories described in this paper complement those documented in our related paper [85] which presented data focused very specifically on the Wellbeing Centre and its organisational response to the pandemic. That paper covered specific harm reduction services such as naloxone distribution, and generic supports such as the expansion of virtual support. This current paper takes a wider perspective, by drawing on data that provide insight into the city centre response to COVID-19 more broadly. In this paper we have discussed: the impact of COVID-19 on mental health and use of drugs and alcohol; the nature of sector-wide organisational and city centre responses to those with multiple and complex needs; and the opportunities presented to extend partnership working and greatly enhance the accessibility and timeliness of harm reduction services. We integrate views from people with lived/living experience regarding the success of such responses in meeting their needs.

Our data show that multiple risks coalesced for individuals over the initial weeks of the pandemic, and the country and city’s response. As warned of by authors such as Wakeman, Green and Rich (2020) [93], overdose risks are increased in pandemic situations due to a perfect storm of: supply side pressures (such as reduced access to opioids which can reduce tolerance); compensation using other substances including alcohol and benzodiazepines which potentiate risk by being unpredictable and riskier (e.g. not responsive to naloxone use, unknown type, non-beverage alcohol); increased risks associated with people injecting alone; and reduction in access to mainstream health, social, and associated supports for treatment, mutual aid fellowship groups, and harm reduction. In the USA, it was reported, for example, that abstinence-based recovery fellowships initially at least ‘shuttered’ [94]. While some essential services, public service or wider, contracted or disappeared, others sprang up to fill the holes. If services close, significant additional risk can be created for those that need them.

This rapid case study reveals a mix of practices and responses. Through participant accounts, particularly those of people with lived/living experience, we are provided with a view into the desperate circumstances that were faced through the complete withdrawal/shuttering of many ‘mainstream’ services. Despite some participants reporting that these had traditionally been largely unresponsive to their needs, or judgemental towards them, they were still missed when they closed. It is well documented that people who experience homelessness/with substance use challenges often have difficulty trusting professionals and services. They may struggle to engage with available support for a range of reasons, including bad previous experiences, and being judged or discriminated against [32]. Due to the myriad of challenges of managing everyday life, healthcare cannot always be prioritised [95]. Our data provide examples of this, both before and during the pandemic. We also know that people experiencing homelessness are more likely to experience mental health problems than the general population [8], again for a range of reasons including a lack of appropriate and responsive primary and secondary care [4, 8]. Participants with lived/living experience in our study described either not being registered with a GP, or not attending their GP, due to feeling judged or discriminated against [4, 96, 97].

As described in the introduction, inclusion health and equity-oriented approaches have utility in helping to radically improve services for people with complex and challenging lives. Both highlight the importance of understanding contexts of substance use-related harms, and the need for cultural safety and violence- and trauma-informed care [89, 98]. They place emphasis on the broader social and structural conditions of people’s lives, including poverty, exclusion, racism, sexism, and criminalisation. MacKinnon et al. (2020) [89], for example, emphasise that care givers should focus on how services are experienced, with active reflection on provider positions of power, privilege, and dominant norms within the healthcare system, which can create exclusion.

We would argue that the organisational responses to COVID-19 described in this paper reflect these approaches. For example, the outreach health responses were focused on taking services to people, rather than expecting people to attend general clinic settings, recognising the multiple barriers to care that are commonly experienced. Indeed, care was put in place around the person, ensuring that a wide range of health and social needs were met in an integrated way. The desire to reduce a range of harms was a major driver in changing practices and policies over the weeks of the lockdown. There was a focus on building on trusted relationships that already existed in familiar settings, such as the Wellbeing Centre, that were viewed by those using services as ‘safe’. People who were at risk of harms were actively sought out, using organisations and staff that they were closest to.

As Lago et al. (2017) [99] argue, trust often requires more risk for people who are marginalised by mainstream cultures, including health and social services: trust must not be assumed when providing any service but must instead be gained through the building of safe relationships. Indeed, Teck and Baldacchino (2020) [100] discuss the importance of trust as a driver for the success of public health interventions. In our case study setting, it was a priority to adapt and ‘join up’ gaps between services, which Teck and Baldacchino highlight as necessary: “*To use the analogy of a plant growing around obstructions to reach the sunshine and thrive, agile and accountable governance and management structures, strong partnership working and culturally informed practices can contribute to the implementation of an effective COVID-19 containment strategy”* [100; p. 1276]. As Gibson (2020) [75] highlighted in describing the city centre partnership work, *“by moving healthcare away from behind clinic walls and taking it out to the people, by walking with patients side by side, by us all working together—we can be more than the sum of our parts”.*

The pandemic provided opportunities for more focused partnership and coordinated working in the city centre, and enhanced and immediate communication across the sector, where competition between organisations was previously commonplace. This aligns with guidance on meeting the needs of people with multiple and complex needs, including with drug and alcohol problems [6], and specific COVID-19 guidance on strengthening partnerships between harm reduction and wider services such as housing and pharmacy providers [46]. These responses were not put in place without challenge, but those advocating for the immediate need for reduced administrative barriers were successful in persuading senior leaders in a range of organisations to adapt and allow change.

In terms of harm reduction most specifically, as Wakeman et al. (2020) [93] highlight, the pandemic acted as a catalyst to create opportunities to bring more people into medically-assisted substance use treatment. These treatments are underutilised and Scotland, like many other nations, struggles to engage many of those who could benefit in treatment such as OST. This is for a range of reasons, including long waits to access treatment after first presentation and the need for multiple appointments. In discussing the required response to the pandemic, Khatri and Perrone (2020) emphasise the importance of providing innovative and low threshold pathways to treatment for new patients, while keeping existing clients engaged [101]. Our data describes people who were opiate dependent considering, and coming into, treatment for the first time because access to supplies of street drugs were uncertain or restricted, and because of lower threshold access facilitated by the adjustments made to OST [85].

Clearly, when mainstream health and addictions service close due to COVID-19 restrictions, there are substantial disruptions to care that place individuals who use these services at even higher risk of a range of harms than usual. As Vasylyeva and colleagues have stressed, COVID-19 is likely to disproportionally affect people who use drugs, not only due to a high prevalence of co-morbidities, but also due to criminalisation, stigmatisation, and a wide range of social and economic challenges that make adhering to quarantine, social distancing, and self-isolating guidelines very difficult if not impossible [92]. Alexander et al. (2020) [94] have discussed the requirement of unprecedented planning and support to limit this disruption and have emphasised the importance of new partnerships, use of technology, and the dismantling of antiquated regulations. The work profiled in our study is an example of the rapid response Wakeman et al. (2020) [93] call for.

### Implications for policy and practice

We know that COVID-19 is illuminating disparities between those who are able to follow guidance in order to prevent infection and transmision and those who cannot for a range of reasons, including lacking the resources to do so such as not having a home [102]. Harm Reduction International published a statement on 11 November 2020 stating that the COVID-19 pandemic has seen unprecedented and ‘long-awaited’ adaptations across the world with regards to harm reduction expansion. In particular, expanded take-home capacities for OST for longer periods, and less restrictive initiation procedures, have been highlighted as both feasible and beneficial [46]. The need for low threshold community settings for the distribution of harm reduction commodities [45], as described in our case study setting, is also crucial. Wakeman et al. (2020) [93] highlight that crisis can lead to opportunity and describe the importance of immediate scale up of availability and outreach to encourage those with opiate disorders to engage with OST as part of the COVID-19 pandemic response, something that should subsequently avert overdose deaths. Furthermore, continuation of the scaling-up of harm reduction strategies after the pandemic abates should be priorised to urgently address the tragic escalation of drug-related deaths in the UK and other nations. Unfortunately, at the time of writing in seems that some of the COVID-19 related better practice has slipped back to ‘business as usual’. However, while countries such as the US have allowed increased flexibility concerning initiation onto OST via non-face-to-face methods, for example, Scotland must make treatment far more responsive and attractive, something that is being led by our national Drug Deaths Taskforce with a wide range of partners [103].

It is encouraging that the Scottish Government recognises that health care needs to be different post COVID-19, and notes the importance of reducing health inequalities: *“We will ensure the health and social care support system is focused on reducing health inequalities”*, and specifically state the need to: *“strengthen relationship-based approaches, and provision of support to those who might be missing (e.g. not using virtual methods, or who do not attend routine appointments)”* [104; p. 7]*.* However, it is important to stress that much of the activity captured in this case study was done outside of usual practice structures and settings because of leadership in particular organisations with shared ethos and values that drove immediate action to try to save lives and reduce harm. It is therefore essential for those leading and managing health services to look carefully at how administration of services can impede relationship-based approaches and restrict ‘on the ground’ partnerships between sectors and professionals. Competitive tendering is one example of this, which we understand has been stopped in the city, at least temporarily, because of a recognition of the damage that this does to the ethos as well as practice of partnership and coordination. We would strongly recommend these approaches are not returned to.

One of the key learning points from this case study is that leadership was and needs to be enacted at all levels of organisations. Engaged leadership of those who are closest to the ‘front-line’ of care is both required, and must be supported by others, in order to deliver change needed ‘on the ground’. Such leadership can create a change in the culture of care where stigma is directly challenged and respectful relationships role modeled for others. The importance of culture and leadership in changing healthcare and wider services and supports for people who are homeless has been underscored by a recently conducted realist review [98]. This review recommends the need for services to be funded using multi-year funding cycles to address the current fragmentation and commissioning culture that can damage an integrated response for this vulnerable group. As Alexander et al. (2020) reminded us during the initial throes of this pandemic: *“The greatest strength of the treatment system has always been compassion and care for the most vulnerable—qualities needed now more than ever”* [94, p. 2]. This case study has shown these qualities to be alive and well in Scotland. To be sustained they need to be valued, considered as best practice locally as well as nationally, and to receive structural support from senior healthcare managers and leaders including sustainable funding.

### Strengths and limitations of the study

We have reflected in detail on strengths and limitations of this study in our linked paper [85]. Due to an existing strong partnership between researchers and the case study service organisation, we were able to conduct in person socially distanced data collection with people using services during the height of the pandemic. Use of different participant viewpoints provides a rich picture of this intense period. In addition, all lived/living experience interviews were conducted by a community researcher who also had a role in the service as a peer support worker (JD). Peer research is advantageous as it can lead to more meaningful and rich data [105]. The main limitation is the smaller number of women participants due to the pragmatic sampling approach and short window for data collection, and the fact that only one case study site was possible due to the unfunded nature of the research. Flyvberg (2006), however, argues that the findings from well-conducted single-case studies can be generalised [106].

## Conclusions

The COVID-19 pandemic has both highlighted and will exacerbate a range of health and social inequalities across the world, impacting most harshly on those with existing health conditions and experiencing socio-economic hardship [92]. This rapid case study has described the significant impact of the COVID-19 pandemic on a group of people experiencing homelessness and problem substance use within one city centre in Scotland and provides a lens on service responses. Few studies to date have focused on this population and sector during the COVID-19 pandemic period. We have highlighted a number of significant lessons coming from this ‘path-breaking’ event that can inform the international and ongoing response to this pandemic for decision makers, service providers, and advocacy and peer organisations working in the field. We have articulated the relevance of inclusion health and equity-orientated approaches to informing responses to COVID-19 in meeting the needs of people who experience homelessness with drug or alcohol concerns. It is vital to build rapidly on early successes that were motivated both by compassion and care for those vulnerable to harms, and the desire to provide high quality evidence-based harm reduction services.

## Supplementary Information

**Additional file 1.** Glossary of Scottish terms

**Additional file 2.** Glossary of terms used

**Additional file 3.** List of abbreviations

## Data Availability

The datasets generated and/or analysed during the current study are not publicly available because study data were initially generated to evaluate the response of one service to the COVID-19 pandemic. Individual privacy could be compromised if the dataset is shared due to the small sample involved and the fact that the city and service is named.
