# Supplementary material for: “You know, we can change the services to suit the circumstances of what is happening in the world”: a rapid case study of the COVID-19 response across city centre homelessness and health services in Edinburgh, Scotland
Source: Harm Reduct J. 2021 Jun 12;18:64. doi: 10.1186/s12954-021-00508-1 (PMC8197599; doi:10.1186/s12954-021-00508-1)
Supplement: Supplementary file 1 — Additional file 1. Glossary of Scottish terms [file 12954_2021_508_MOESM1_ESM.docx]

**Additional File 1. Glossary of Scottish terms**

Ain - own

Cannae – can’t

Dae – do

Didnae – didn’t

Dinnae – don’t

Fae - from

Gi’ – give

Gonnae – going to/gonna

Havenae – haven’t

Isnae – isn’t

Ken – know

Mare - more

Nae – no

Naebody – nobody

Naewhere – nowhere

No – not

Oot - out

Tae – to

Wasnae – wasn’t

Wee – small/little

Wi’ – with

Wouldnae – wouldn’t

Yous – you (plural)
