# Supplementary material for: “You know, we can change the services to suit the circumstances of what is happening in the world”: a rapid case study of the COVID-19 response across city centre homelessness and health services in Edinburgh, Scotland
Source: Harm Reduct J. 2021 Jun 12;18:64. doi: 10.1186/s12954-021-00508-1 (PMC8197599; doi:10.1186/s12954-021-00508-1)
Supplement: Supplementary file 2 — Additional file 2. Glossary of terms used [file 12954_2021_508_MOESM2_ESM.docx]

**Additional file 2 – Glossary of terms used**

**Commissioning**

The process of planning, agreeing and funding health services. In Scotland, substance use services are commissioned by Alcohol and Drug Partnerships.

**National Health Service (NHS)**

Publically funded healthcare system in the UK, which is free at the point of entry.

**Non-beverage alcohol**

Alcohol products that are not intended for human consumption, such as hand sanitisers, aftershave and anti-freeze.

**Shielding**

A term used during the COVID-19 pandemic to describe the situation where those deemed high risk should protect themselves by not leaving their homes and minimising all face-to-face contact.

**Third sector services**

Organisations that are non-governmental and non-profit making, such as charities. They can also be known as ‘not for profit’.
