# Supplementary material for: “You know, we can change the services to suit the circumstances of what is happening in the world”: a rapid case study of the COVID-19 response across city centre homelessness and health services in Edinburgh, Scotland
Source: Harm Reduct J. 2021 Jun 12;18:64. doi: 10.1186/s12954-021-00508-1 (PMC8197599; doi:10.1186/s12954-021-00508-1)
Supplement: Supplementary file 3 — Additional file 3. List of abbreviations [file 12954_2021_508_MOESM3_ESM.docx]

**Additional File 3. List of abbreviations**

A&E – accident and emergency

COPD - chronic obstructive pulmonary disease

CPN - Community Psychiatric Nurse

GP – General practitioner

IEP - injecting equipment provision

MAP(s) - Managed Alcohol Programme(s)

NHS - National Health Service

OST - opioid substitution treatment

PPE - personal protective equipment

SHAAP - Scottish Health Action on Alcohol Problems
